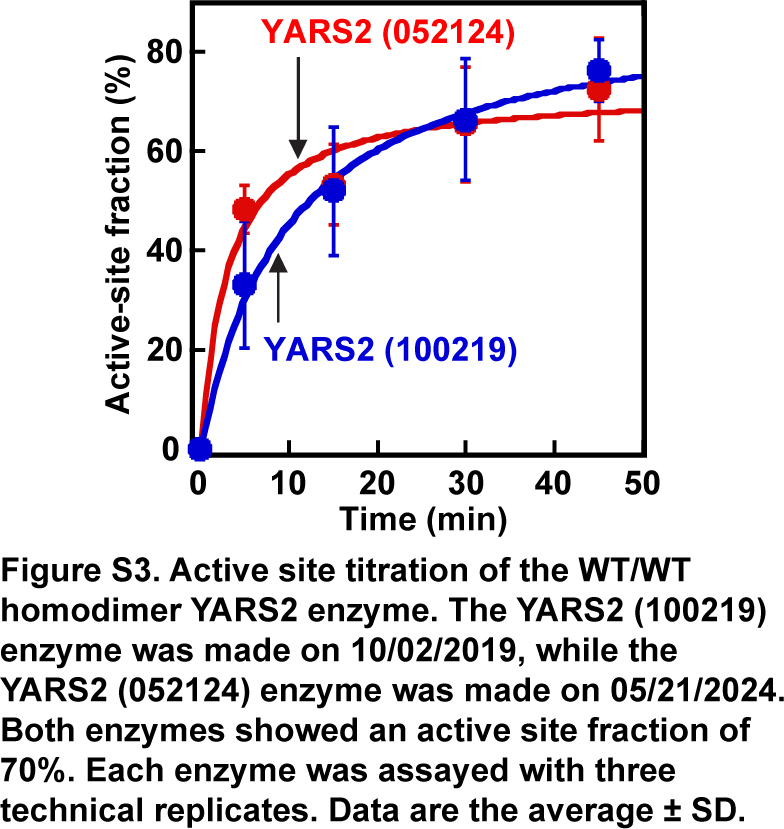# A kinetic model for compound heterozygous pathogenic variants in Tyrosyl-tRNA synthetase gene *YARS2*-Associated neonatal phenotype

**DOI:** 10.1016/j.jbc.2024.108092

**Published:** 2024-12-13

**Authors:** Thomas Christian, Sunita Maharjan, Sitao Yin, Yuka Yamaki, Isao Masuda, Fenglin Li, Colleen Muraresku, Sheila Clever, Rebecca D. Ganetzky, Ya-Ming Hou

**Affiliations:** 1 Department of Biochemistry and Molecular Biology, Thomas Jefferson University, Philadelphia, USA; 2 Department of Biochemistry and Biophysics, University of Pennsylvania, Philadelphia, USA; 3 Department of Pediatrics, Perelman School of Medicine, Philadelphia, Pennsylvania, USA; 4 Mitochondrial Medicine Frontier Program, Human Genetics Division, CHOP, Children’s Hospital of Philadelphia, Philadelphia, Pennsylvania, USA

**Keywords:** mt-TyrRS, mt-tRNA^Tyr^, MLASA, homodimer, heterodimer

## Abstract

Human genetic disorders are often caused by mutations of compound heterozygosity, where each allele of the mutant gene harbors a different genetic lesion. However, studies of such mutations are hampered due to the lack of an appropriate model. Here we describe a kinetic model of compound heterozygous variants in an obligate enzyme dimer that contains one mutation in one monomer and the other mutation in the second monomer. This enzyme is encoded by human *YARS2* for mitochondrial tyrosyl-tRNA synthetase (mt-TyrRS), which aminoacylates tyrosine to mt-tRNA^Tyr^. *YARS2* is a member of the genes for mt-aminoacyl-tRNA synthetases, where pathogenic mutations present limited correlation between disease severity and enzyme activity. We identify a pair of compound heterozygous variants in *YARS2* that is associated with neonatal fatality. We show that, while each mutation causes a minor-to-modest defect in aminoacylation in the homodimer of mt-TyrRS, the two mutations *in trans* synergistically reduce the enzyme activity to a greater effect. This kinetic model thus accurately recapitulates the disease severity, emphasizing its utility to study *YARS2* mutations and its potential for generalization to other diseases with compound heterozygous mutations.

Compound heterozygosity is one of the most common inheritance types for recessive diseases in human genetic disorders of Mendelian traits (1, 2). Such inheritance occurs in an individual where the two alleles of the disease-associated gene have different pathogenic variants. This genetic heterogeneity is challenging to assess the pathogenicity of each variant, especially when each is associated with a different monomer in an obligate enzyme dimer, raising the question of how each mutated copy of the protein interacts with the other copy to affect the enzyme activity. Current studies of compound heterozygosity have addressed only one mutation at a time, usually modeled as homozygous variants. This is the case in enzymes of the aminoacyl-tRNA synthetase (ARS) family (3, 4, 5, 6).

ARS enzymes are essential for cell viability. Each enzyme typically activates one of the canonical amino acids and charges it to the cognate tRNAs to generate aminoacyl-tRNAs for delivering the amino acid to the corresponding mRNA codons during protein synthesis (7). Structurally, ARSs are divided into two classes—the class I structure uses the Rossmann-fold to build the active site in eight monomeric and two dimeric enzymes, while the class II structure uses three non-Rossmann motifs to build the active site in ten oligomeric enzymes (7). Clinically, all ARSs have been implicated in human diseases (8, 9, 10). Given their predominance in oligomeric structures, compound heterozygosity is an important challenge to our understanding of ARS-associated diseases.

The human nuclear genome encodes a set of ARSs dedicated for aminoacylation in the cytosol, and a separate set for aminoacylation in mitochondria (the ARS2 enzymes) (7). Pathogenic variants in ARS2s are increasingly recognized as a clinically important subclass of mitochondrial diseases (11). Notably, the functional state of each ARS2 directly impacts the activity of its mt-tRNA in mitochondrial translation, where each mitochondria-encoded protein is an essential component of the electron transport chain that synthesizes ATP. Diseases resulting from ARS2 deficiencies are pleiotropic and present a broad spectrum of clinical features (12). Yet, there is limited correlation between enzyme activity and disease severity (12), primarily due to the lack of a kinetic model to address compound heterozygosity, thus preventing early treatment and counseling to reduce morbidity or mortality.

Here we present a case of compound heterozygosity associated with mitochondrial Tyr-specific *YARS2*. Disease resulting from pathogenic variants in *YARS2* exemplifies the clinical challenges of ARS2 deficiencies. While biallelic loss-of-function variants in *YARS2* are associated with the MLASA (myopathy, lactic acidosis, and sideroblastic anemia) syndrome (13), the spectrum of disease, including age of onset and presentation features, is broad (13, 14, 15). Also, the reported impact of variants on the enzyme activity does not correlate with disease severity (5). Indeed, while the *YARS2* enzyme is an obligate dimer of the class I structure (16), studies of subjects with compound heterozygous variants so far modeled each variant as a homodimer (5, 13, 17). The compound heterozygosity we report here is associated with severe morbidity, including neonatal lactic acidosis, pulmonary hypertension, and ultimately infantile fatality, representing the most clinically severe case of *YARS2*-related disease to date. To understand this disease severity, we developed a heterodimer model, placing one mutation in one monomer and the other mutation in a second monomer, thus evaluating both mutations at the same time. We show that, while each variant in a homodimer has a minor-to-modest effect on the enzyme activity, the two variants together profoundly reduce the enzyme activity. This study demonstrates the importance of heterodimer modeling of *YARS2* variants in a framework that is generalizable to other compound heterozygosity cases involving enzymes of obligate dimers or oligomers.

## Results

### Patient presentation

**Figure 1:**
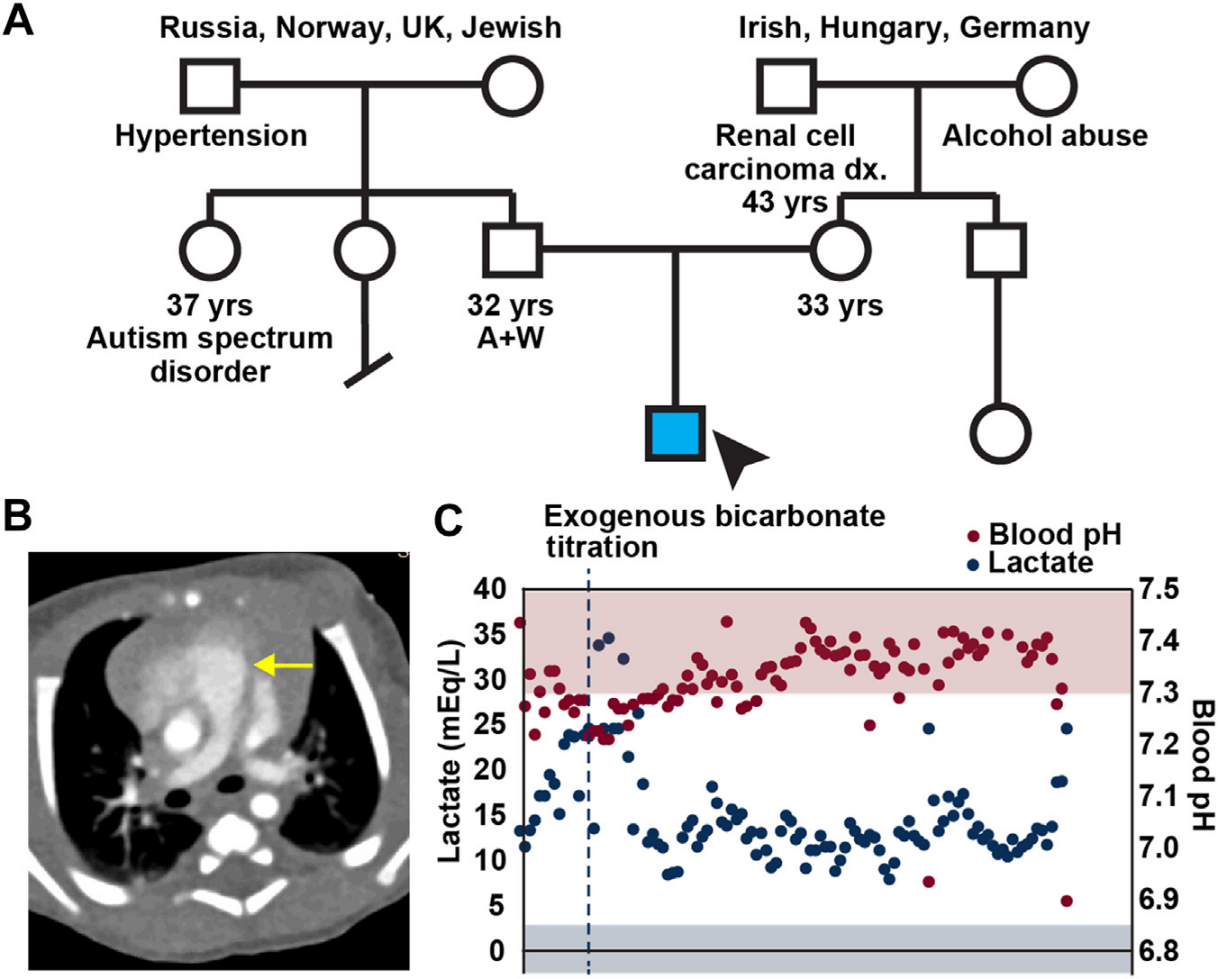
**The subject**. *A*, pedigree of the proband. *B*, chest CT angiogram showed right-sided cardiac enlargement and dilation of the main pulmonary artery (*arrow*), indicating pulmonary hypertension. *C*, patient’s lactate (*blue*) and blood pH (*red*) from 4 weeks of age until demise at 8 weeks. Shaded rectangles indicate normal reference ranges. Exogenous bicarbonate was initiated to attempt to buffer lactate (*arrow*).

The patient was the first child of a non-consanguineous union with a 32-year-old mother who was gravida 1 (G1: the first pregnancy) and para 0 (P0: not yet given birth) (Fig. 1*A*). The patient was of mixed European and Ashkenazi Jewish ancestry. Through an un-complicated pregnancy, he was born at 40 weeks by spontaneous vaginal delivery. Birth weight was 3.66 kg (50th percentile) and he was discharged from the well-baby nursery on day 2. On day 3 of life, however, he had a heart murmur; and a full cardiac evaluation performed at 4 weeks of age revealed pulmonary hypertension and failure to gain weight. Over the next week, he had worsening oral intake and lethargy. The echocardiogram showed progression of pulmonary hypertension, which was confirmed by CT angiogram (Fig. 1*B*). He underwent a lung biopsy, which showed pulmonary interstitial glycogenosis. He was admitted for management of pulmonary hypertension and was tested for blood gas, which showed an initial venous pH of 7.158 (normal level (nl): 7.35–7.45) and a venous lactic acid level of 13.74 mEq/L (nl: 0.5–2.2 mEq/L). Over the next 4 weeks, his lactic acidosis was buffered with exogenous sodium bicarbonate. However, at eight weeks, with his lactate peaked at 35 mEq/L (Fig. 1*C*), resulting in intractable metabolic acidosis and systemic hypotension, he passed away. Clinical whole exome sequencing analysis revealed biallelic variants *in trans* in *YARS2*: NM 001040438.2: c.553T>C (p.F185L, paternally inherited) and NM 001040436.2: c.792_794delAGA (p.E264del, maternally inherited). Both were classified as likely pathogenic variants.

### Sequence and structure-guided analysis

**Figure 2:**
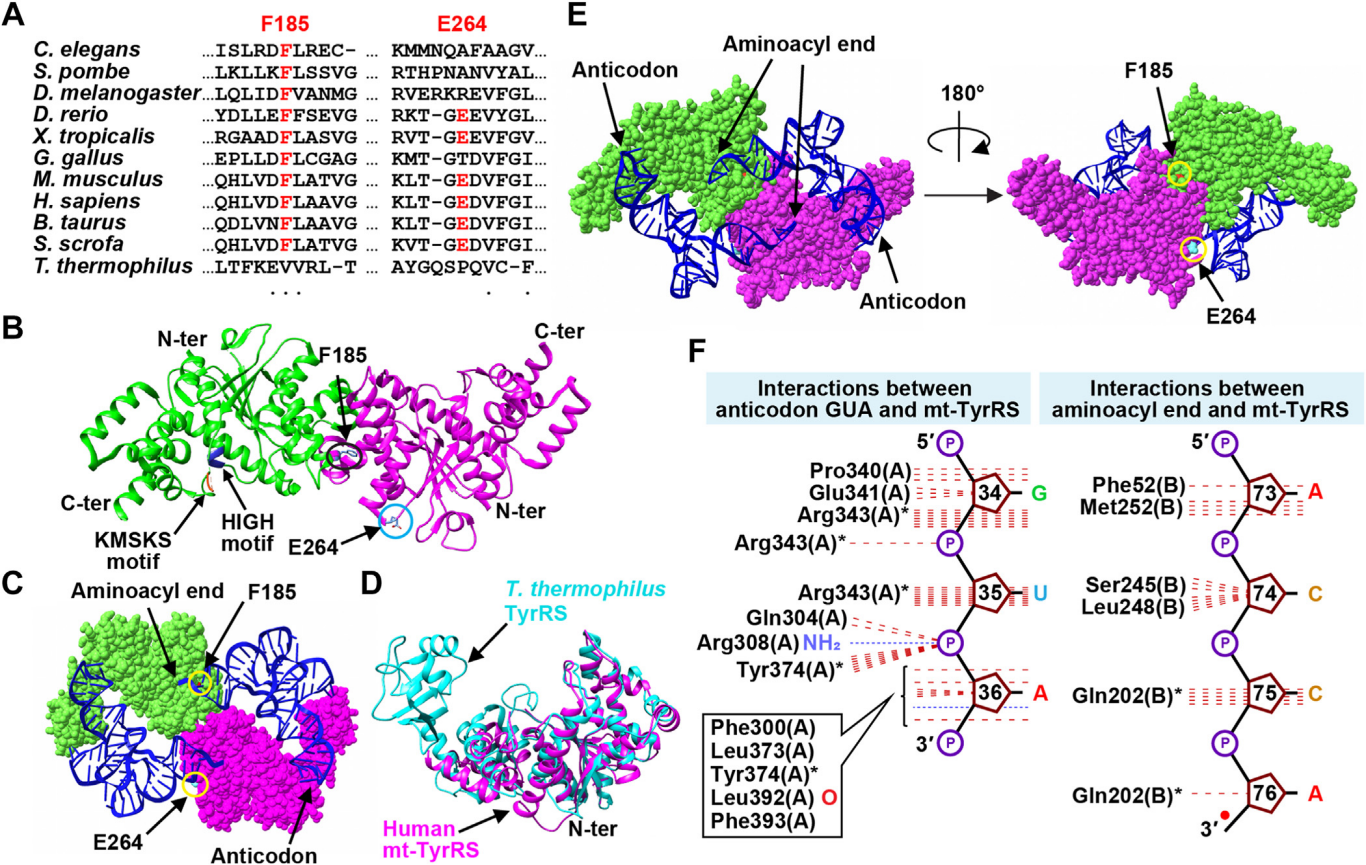
**Structural modeling of mt-TyrRS**. *A*, sequence alignment of mt-TyrRS from *C. elegans* to human, showing positions of F185 and E264, along with *Tt* TyrRS. *B*, crystal structure of the human mt-TyrRS dimer (PDB:2PID), showing the N- and C-termini of each monomer. A truncated mt-TyrRS lacking residues after L373 was used in the structure. Highlighted are the class I catalytic motifs HIGH and KMSKS of the green monomer, and the F185 and E264 residues of the magenta monomer. *C*, crystal structure of *Tt* TyrRS with the *Tt* tRNA^Tyr^ transcript (PDB:1H3E), marking F185 and E264. *D*, structural alignment of human mt-TyrRS (*magenta*, 2PID) with *Tt* TyrRS (*cyan*, 1H3E). *E*, the AlphaFold-modeled structure of human mt-TyrRS (2PID) bound to *Tt* tRNA^Tyr^ (from 1H3E). *F*, H-bonding interactions of the modeled complex in *E* with the GUA anticodon and the CCA end of tRNA^Tyr^ in the canonical tRNA nucleotide positions.

In a multi-sequence alignment of *YARS2* enzymes (Fig. 2*A*), each mutation conferred a distinct biochemical feature. While F185L replaced a strictly conserved aromatic side chain among eukaryotic mitochondrial sequences, ΔE264 removed a negatively charged side chain. In the crystal structure of mt-TyrRS (16), F185 is in the connective polypeptide 1, connecting the N- and C-terminal cores of the aminoacylation domain, while E264 is in the C-terminal core, facing the solvent (Fig. 2*B*). In the related tRNA^Tyr^-bound *Thermus thermophilus* (*Tt*) TyrRS structure (18), both F185 and E264 are near the tRNA 3′-end but are not in direct contact (Fig. 2*C*). Notably, while two tRNAs are bound to one enzyme dimer, each tRNA binds across the dimer interface, starting from the anticodon-end in contact with one monomer and crossing over to the other monomer to insert the aminoacyl end to the active site (18). As both monomers are required for each tRNA to stably interact with *Tt* TyrRS, this binding mode illustrates the essentiality of the dimer as the functional unit of the enzyme. Notably, mt-TyrRS currently has no tRNA-bound structure. Due to its low sequence homology with *Tt* TyrRS (identity 27%, similarity 42%), we used AlphaFold to model mt-TyrRS in complex with tRNA. This modeling revealed that mt-TyrRS aligns well with *Tt* TyrRS and that it adopts a tRNA-bound structure closely similar to that of the *Tt* complex (Fig. 2, *D* and *E*), showing the conserved enzyme interface with the tRNA anticodon GUA in one monomer and with the 3′ CCA sequence in the other monomer (18) (Fig. 2*F*), which validates the binding of each tRNA across the dimer interface.

### Kinetic analysis of homodimers

**Figure 3:**
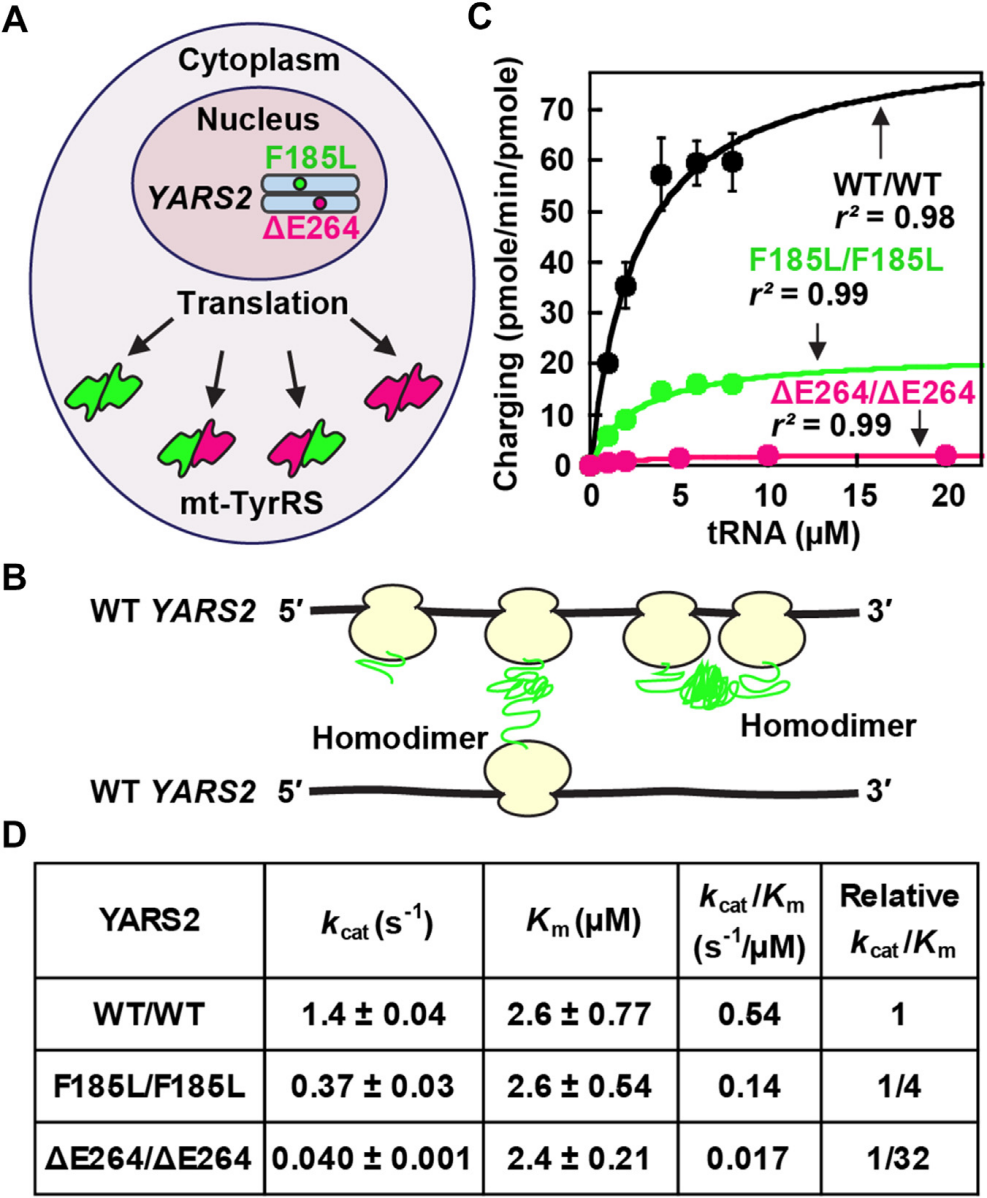
**Aminoacylation of homodimers**. *A*, protein expression of compound heterozygosity of the subject, showing the 1:1:2 ratio of the homodimers F185L/F185L (*green*) and ΔE264/ΔE264 (*magenta*) to the heterodimer F185L-ΔE264. *B*, the nascent chain of an mt-TyrRS monomer of one ribosome can associate with the nascent chain of an adjacent ribosome on the same or a different mRNA to form a homodimer (19). *C*, Michaelis-Menten plots of the three homodimers WT/WT, F185L/F185L, and ΔE264/ΔE264, showing the *r*^2^ of each. Error bars are shown for the WT/WT but are invisible for the two variants. *D*, kinetic parameters of each homodimer. Values are the average ± SD, N = 3 to 6.

We determined how each mutation would affect the activity of mt-TyrRS in a homodimer. If the two *YARS2* alleles of the subject, one each from a parent, were equally and independently expressed for protein synthesis and were of similar protein stability, we would expect a molar ratio of each homodimer relative to the heterodimer at 1:1:2 (Fig. 3*A*). We thus expressed and purified three homodimers—WT/WT, F185L/F185L, and ΔE264/ΔE264, each from a plasmid-borne single gene in *Escherichia coli* (16). The association of two monomers to form a homodimer can occur spontaneously during protein synthesis when the nascent chain of a ribosome on an mRNA directly interacts with the nascent chain of an adjacent ribosome on the same or a different mRNA (Fig. 3*B*) (19).

We assessed the aminoacylation activity of each homodimer by kinetic analysis in steady-state conditions (Fig. 3, *C* and *D*), where tRNA was in molar excess to each enzyme to allow multiple turnovers. The tRNA substrate was generated by IVT (*in vitro* transcription) of the sequence of *E. coli* tRNA^Tyr^, based on the report that *E. coli* tRNAs are substrates for bovine ARS2 enzymes (20) and on the finding that the IVT yield of *E. coli* tRNA^Tyr^ was much higher relative to mt-tRNA^Tyr^. The Michaelis-Menten plots of the WT/WT homodimer and the derived kinetic parameters *k*_cat_, *K*_m_, and *k*_cat_/*K*_m_ were similar to those of the published data of mt-TyrRS (21, 22) and were consistent with parameters of ARS enzymes in general (23, 24, 25), validating that the reagents and assay conditions were suitable. Notably, the F185L/F185L homodimer retained kinetic parameters close to those of WT/WT, showing a minor 4-fold loss of *k*_cat_ that drove the loss of *k*_cat_/*K*_m_. In contrast, the ΔE264/ΔE264 homodimer had a greater loss of *k*_cat_ by ∼30-fold, which also drove the loss of *k*_cat_/*K*_m_. Thus, both variants had deficiency in *k*_cat_, leading to losses of the catalytic efficiency *k*_cat_/*K*_m_.

### Kinetic analysis of heterodimers

**Figure 4:**
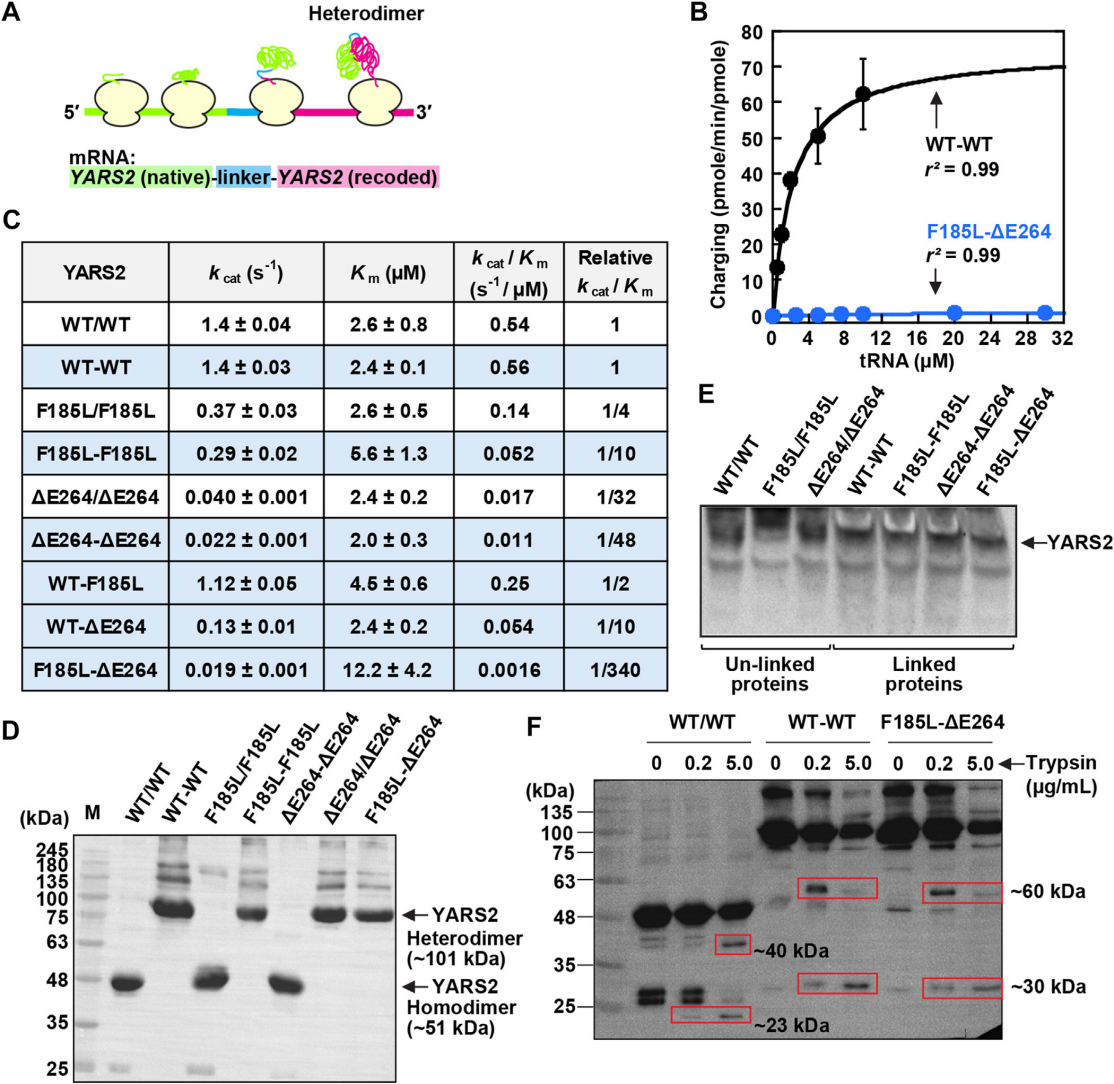
**Aminoacylation of heterodimers.***A*, assembly of a heterodimer *via* a covalent link of two monomers as translated by a ribosome based on one copy of *YARS2* in the native sequence (*green*), followed by the linker (*cyan*), and the recoded sequence (*magenta*). *B*, Michaelis-Menten plots of WT-WT and F185L-ΔE264, showing the *r*^2^ of each. *C*, kinetic parameters of each homodimer *vs.* heterodimer, showing the fold-decrease in *k*_cat_/*K*_m_ relative to WT/WT. Individual values are the average ± SD (N = 4–6). *D*, Western blot of an SDS-PAGE of homo- and heterodimers, where the markers were superimposed from the image of a pre-stained gel. *E*, Western blot of a native PAGE of unlinked and linked proteins, marking the YARS2 protein by an arrow, which was recognized by the α-His antibody (as shown) and the α-mt-TyrRS antibody (not shown). The *lower band* was recognized only by the α-His antibody, indicating that it is a co-purified contaminant. The different migration profile of F185L/F185L could be due to perturbation of the dimer interface when F185L was in an unlinked homodimer. *F*, Western blot analysis of limited trypsin digestion of WT/WT, WT-WT, and F185L-ΔE264, showing degradation products in *red boxes*.

We next determined how the two mutations in compound heterozygosity affected the activity of mt-TyrRS. As these two mutations were *in trans* to each other, we developed a heterodimer model to place one mutation in one monomer and the other mutation in the second monomer. Notably, while co- or post-translational assembly of heterodimers have been done (26, 27, 28, 29), they are nontrivial—typically starting with expression of each homodimer, followed by separation of each into subunits, mixing differently tagged subunits, and isolation of heterodimers by two steps of affinity purification. These methods cannot control the stoichiometry of each monomer, due to the differential cellular expression of each. We addressed this limitation by expressing two copies of *YARS2* that were genetically linked, in which one copy encoded the natural sequence of the gene while the second “recoded” the gene using synonymous codons to maintain the same amino acid sequence (Fig. 4*A*). While the linker conferred flexibility (30), the sufficiently different sequences of the two genes allowed the introduction of F185L to one copy and ΔE264 to the second copy. During protein synthesis, the nascent chain of each ribosome would spontaneously fold into a covalently linked heterodimer (Fig. 4*A*).

**Supplemental Figure S1:**
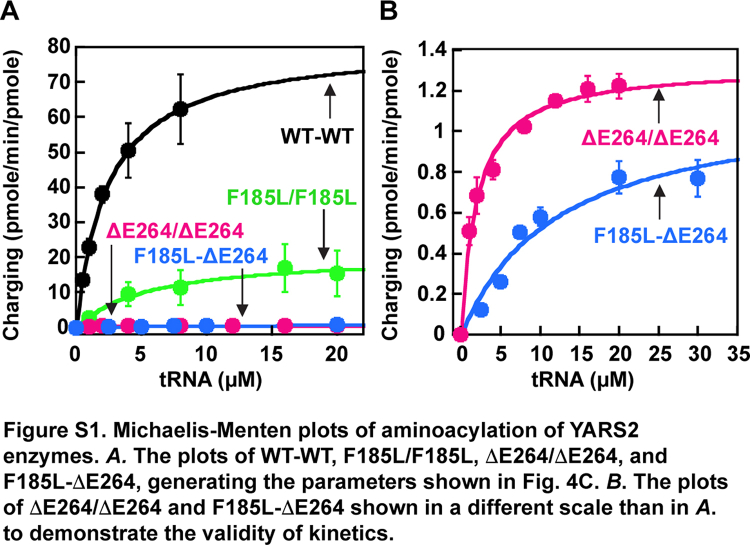


Aminoacylation assays showed that the covalently linked WT-WT heterodimer had virtually identical kinetic parameters as those of the unlinked WT/WT homodimer (Fig. 4, *B* and *C*), validating that the two distinctly coded monomers were associated as a functional unit of the native dimer and that the linker does not interfere with tRNA binding. Indeed, the kinetic parameters of two additional heterodimers were also close to those of the respective homodimers (*i.e.*, heterodimer F185L-F185L vs. homodimer F185L/F185L; heterodimer ΔE264-ΔE264 vs. homodimer ΔE264/ΔE264, Figs. 4*C* and S1, *A* and *B*). We thus compared the kinetic parameters of the heterodimer F185L-ΔE264 vs. those of the WT-WT heterodimer, showing a substantial loss of *k*_cat_/*K*_m_ by 340-fold of the mutant. This loss was much greater than the combined effect of the two heterodimers ((10-fold of F185L-F185L) + (48-fold of ΔE264-ΔE264) = 58-fold). If the two mutations were independent of each other, their combined effect would have been additive (31). Thus, the two mutations were inter-related *in trans*, creating a synergistic effect. We also found that the ΔE264 mutation has a dominant-negative effect. The heterodimer WT-ΔE264 had a 10-fold loss of *k*_cat_/*K*_m_ relative to the WT-WT heterodimer (Fig. 4*C*), indicating that it prevented the normal activity of the WT enzyme. In contrast, the F185L mutation has no dominant-negative effect, as the WT-F185L enzyme had only a 2-fold loss relative to the WT-WT enzyme.

The substantial loss of *k*_cat_/*K*_m_ of the heterodimer F185L-ΔE264 was not due to changes of the enzyme global structure. While a denaturing SDS-PAGE confirmed that each heterodimer nearly doubled the size of the homodimer (from ∼50–100 kDa, Fig. 4*D*), a native PAGE that assessed protein oligomer structure (32) showed that all enzymes retained the dimer structure (Fig. 4*E*). The similar migration of WT/WT and WT-WT indicates that the linker does not change the dimer structure while that of WT-WT and F185L-ΔE264 indicates that the two mutations do not disrupt the dimer.

**Supplemental Figure S2:**
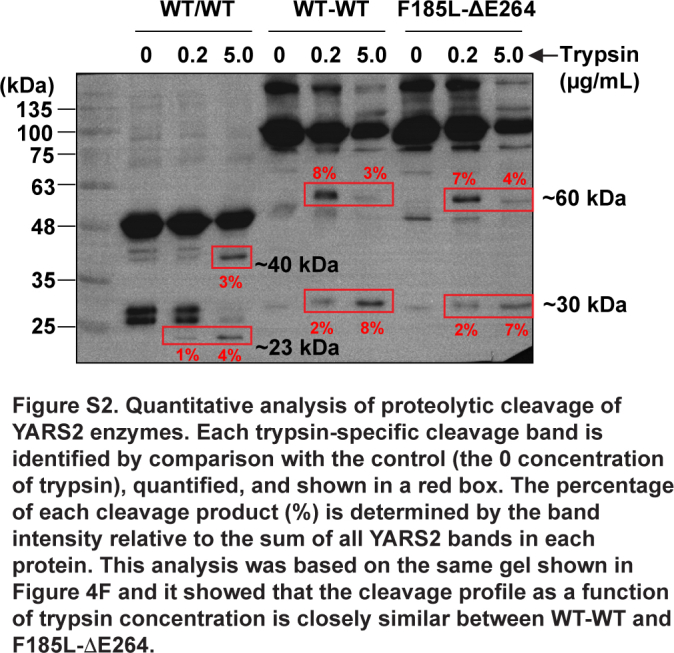


We confirmed that F185L-ΔE264 has a similar global structure as that of the WT/WT and WT-WT enzymes by limited trypsin proteolysis (Fig. 4*F*). Over a fixed incubation time, WT/WT showed sensitivity to trypsin starting at 0.2 μg/ml, producing first a degradation product of 23 kDa, and then enhancing the product with trypsin at 5.0 μg/ml and generating a new product of 40 kDa. This sensitivity was observed with both the WT-WT and the F185L-ΔE264 heterodimers, showing sensitivity starting at 0.2 μg/ml with first the degradation products of 60 kDa and 30 kDa, and then enhancing the 30 kDa product at 5.0 μg/ml. The similar time-dependent sensitivity between WT/WT and WT-WT validated that the linker maintained the global structure of the enzyme. The nearly identical sensitivity between WT-WT and F185L-ΔE264, in both the concentration profile of trypsin (Fig. 4*F*) and the quantitative cleavage as a function of trypsin (Fig. S2), indicated that the mutant heterodimer had retained the structure of the WT and that its kinetic defect was solely due to the two mutations.

## Discussion

*YARS2* is an exemplary model to study compound heterozygosity. In countries with low level of consanguinity, while most *YARS2* variants in populations are compound heterozygous, the clinical information of the disease is largely based on the F52L founder variant of a single ethnic background (13), due to the difficulty of studying compound heterozygosity. As such, most of the compound heterozygosity cases were not reported, except for the single case with the P112R/L208R mutations, which was studied as a homodimer (33). This problem is not unique to *YARS2* but is common to all human genetic diseases, representing a major challenge in modern genetic medicine. Here we present a kinetic model for a pair of compound heterozygous variants of *YARS2* that readily predicts the severity of the disease. In this kinetic model, one variant of the enzyme monomer is covalently linked to the other variant monomer, enabling analysis of the compound heterozygous variants in one obligate heterodimer. While each variant in the obligate dimer causes a minor-to-modest defect in *k*_cat_/*K*_m_ of the enzyme, the two variants *in trans* synergistically generate a much larger effect, which at the loss of 340-fold in *k*_cat_/*K*_m_, is substantially larger than those of other pathogenic *YARS2* variants. For example, aminoacylation kinetics of the F52L variant of *YARS2* was only reduced by 9-fold in *k*_cat_/*K*_m_ as shown in a homodimer model of patients who displayed the MLASA syndrome but lived to ∼20 years of age (13). Thus, the magnitude of the kinetic defect of the F185L-ΔE264 heterodimer closely correlates with the extreme disease severity of the proband, validating the utility of the model to report the genotype-phenotype correlation.

The heterodimer model also reports the nature of the two mutations. Their synergistic effect *in trans* indicates a long-range interaction across the dimer interface in the molecular pathway that transmits the signal of tRNA binding from one end to the other end. This signaling likely starts upon anticodon binding to one monomer, activating dynamic and physical changes of all-atom motions to propagate the changes to the other monomer for aminoacylation. While the WT enzyme efficiently and correctly activates the all-atom motions from one monomer to the other, the two mutations in cooperation with each other likely disrupt the pathway. While preliminary molecular dynamic simulation supports this possibility, a more detailed analysis, as shown in our work of another obligate dimer enzyme (34), is necessary.

The cellular defect of the F185L/ΔE264 variant is likely more complicated. While protein expression of the two alleles of *YARS2* should produce the 1:1:2 ratio of each homodimer vs. the heterodimer (Fig. 3*A*), indicating a 50% representation of the heterodimer to drive the deficiency of the enzyme, this is subject to cellular modifiers of gene expression. Notably, direct assessment of the enzyme activity using the fibroblasts of the patient was unsuccessful, as we detected no clear deficiency in the enzyme activity, protein synthesis, or mitochondrial respiration (not shown). This emphasizes that the cellular manifestation of *YARS2* variants is highly specific to cell types and tissues and that fibroblasts are not suitable as a cell model. Future work will focus on generating a patient-derived iPSC line, along with an isogenic control line, for cell-type-specific differentiation. Of interest are myocytes, as skeletal myopathy is the most notable feature of clinical cases of *YARS2* variants (15, 17), and additionally peripheral neurons, which are affected in most clinical cases of *ARS2* variants (35, 36).

In summary, we present a heterodimer model for a pair of pathogenic variants in compound heterozygosity in *YARS2*-associated neonatal phenotype. This model is built on a construct that co-expresses the two variants in two copies of the gene that are covalently linked. It recapitulates the molecular relationship of the two variants *in trans* without altering the dimer or global structure of the enzyme, and successfully reports the kinetic deficiency of the compound heterozygosity that correlates with the disease severity. These results validate the model to study *YARS2* deficiency and its potential as a template generalizable to other compound heterozygous variants.

## Experimental procedures

### Fibroblast line establishment

The patient has been de-identified. A primary dermal fibroblast cell line was established by CHOP from a 3 mm punch skin biopsy performed post-mortem. Whole exome sequencing at GeneDx confirmed the reported biallelic *YARS2* mutations *in trans*. No other genetic variants were identified.

### Modeling of mt-TyrRS in complex with tRNA^Tyr^

The atomic model of human mt-TyrRS (PDB: 2PID) in complex with tRNA^Tyr^ was made using AlphaFold v3 (37). The structure of tRNA^Tyr^ was from the *Tt* TyrRS-tRNA complex without post-transcriptional modifications (PDB: 1H3E).

### Genetic constructs

The WT/WT homodimer construct, provided by R. Giege (U. Strasbourg), encodes the recombinant human *YARS2*, lacking the mitochondrial targeting sequence but including sequences for the S4-like domain and a C-terminal His-tag. This construct was made in plasmid pQE-70 and was expressed in *E. coli* BL21(DE3). The F185L and ΔE264 mutations were separately generated by QuikChange. The WT-WT heterodimer construct was made by GenScript, where the native *YARS2* was covalently linked to a recoded gene and the cassette was cloned into the BamHI and EcoRV sites of pET30a. The recoded gene harbored nucleotide substitutions at the wobble position of ∼55% of the codons over the entire coding sequence. The F185L-ΔE264 heterodimer was made in the WT-WT construct, where F185L was in the native sequence, while ΔE264 was in the recoded sequence. Protein purification of each enzyme was as described (16). The linker sequence in all heterodimers was: GGGGSGGGG-SGGGGSGGGGS.

### Aminoacylation assays

Steady-state aminoacylation assays with tyrosine were as described (38). The *K*_m_ (tRNA) and *k*_cat_ of each enzyme were derived by fitting data to the Michaelis-Menten equation using the enzyme dimer to calculate the *k*_cat_ but without correcting for the active fraction of the enzyme. Notably, active-site titrations of the WT/WT enzyme for synthesis of the enzyme-bound adenylate (39) showed a consistent value of 70% (Fig. S3), validating the reproducibility of the enzyme purification protocol. Each assay was performed with ≥3 biological replicates.

### PAGE analysis

Denaturing 12% SDS-PAGE of each enzyme (20 μg), as measured by a Bradford assay, was run at room temperature (RT) on a BioRad mini-gel apparatus, while native 8% PAGE (final concentrations of 0.75 M Tris-pH 8.8, 16% Bis-acrylamide, 0.1% ammonium persulfate, 6 μl of TEMED) analysis of each enzyme (20 μg) was run at 4 °C with constant 90 V for 3 h. Both types of gels were processed for Western blot analysis as described (40).

### Limited proteolysis

Each YARS2 protein (20 μg) was digested with three concentrations of trypsin (Sigma, T7409) (0, 0.2, 5 μg/ml) for 5 min at 37 °C (41). These concentrations were selected as optimal from a range of concentrations that were first evaluated for the sensitivity of the WT/WT enzyme to digestion. Digestion was stopped by adding 4× SDS loading buffer (0.2 M Tris-HCl, 0.4 M DTT, 8.0% (w/v) SDS, 6 mM bromophenol blue, 4.3 M glycerol) and incubated at 85 °C for 5 min. The samples were run on a 12% SDS-PAGE, RT, and probed by Western blot analysis.

## Data availability

All data are provided in the manuscript.

## Supporting information

This article contains supporting information.

## Conflict of interest

The authors declare that they have no conflicts of interest with the contents of this article.

## Figures and Tables

**Supplemental Figure S3:**